# The antiviral JNJ-A07 significantly reduces dengue virus transmission by *Aedes aegypti* mosquitoes when delivered via blood-feeding

**DOI:** 10.1126/sciadv.adr8338

**Published:** 2024-11-27

**Authors:** Ana L. Rosales-Rosas, Sara Goossens, Winston Chiu, Atreyee Majumder, Alina Soto, Serge Masyn, Bart Stoops, Lanjiao Wang, Suzanne J. F. Kaptein, Olivia Goethals, Leen Delang

**Affiliations:** ^1^ KU Leuven, Department of Microbiology, Immunology and Transplantation, Rega Institute for Medical Research, Virus-Host Interactions & Therapeutic Approaches (VITA) Research Group, 3000 Leuven, Belgium.; ^2^ KU Leuven, Department of Microbiology, Immunology and Transplantation, Rega Institute for Medical Research, Virology, Antiviral Drug & Vaccine Research Group, 3000 Leuven, Belgium.; ^3^ Janssen R&D, Titusville, NJ, USA.; ^4^ Janssen Global Public Health, Janssen Pharmaceutica NV, Beerse, Belgium.; ^5^ Janssen R&D, Beerse, Belgium.

## Abstract

Dengue virus (DENV) is the most widespread mosquito-borne virus worldwide, but no antiviral therapies are available yet. The pan-serotype DENV inhibitor JNJ-A07 has shown potent activity in a mouse model. It remains unknown whether an antiviral drug ingested by mosquitoes could inhibit virus replication and thus reduce transmission to other hosts. Here, we investigated the antiviral activity of JNJ-A07 when administered in the blood meal to *Aedes aegypti* mosquitoes. JNJ-A07 blocked DENV-2 transmission by the mosquitoes in both pre-exposure and post-exposure settings. In addition, JNJ-A07 remained in the mosquito bodies for 7 days after blood meal. Reductions of DENV systemic infection in the mosquitoes suggested a potential for decreased proportions of DENV outbreaks in a simulated environment when the mosquitoes ingested JNJ-A07 via the blood meal.

## Teaser

JNJ-A07 delivered in the blood meal to *Aedes* mosquitoes reduced DENV transmission and led to potentially less outbreaks in silico.

## INTRODUCTION

Dengue is an infectious disease caused by dengue virus (DENV), one of the most prevalent pathogenic mosquito-borne viruses worldwide (*1*). DENV is a positive-stranded RNA virus that belongs to the Flaviviridae family. Four antigenically distinct serotypes exist, which can all be transmitted to humans by *Aedes* (*Ae.*) mosquitoes and cause disease (*2*). Dengue is endemic in tropical and subtropical regions, including over 125 countries in the world, with the Americas, Southeast Asia, and Western Pacific regions enduring most of the global disease burden (*3*–*5*). However, autochthonous cases and small outbreaks periodically occur in Europe: At the end of 2023, 128 nontravel-associated dengue cases were reported from Italy, France, and Spain (*6*). Currently, an estimated 100 million to 400 million infections occur annually, with 20 to 60% of them presenting symptoms (*7*–*9*). The symptomatic patients can develop clinical manifestations ranging from a mild flu-like illness—characterized by fever, headache, muscle and joint pain, and rashes—to a severe form of the disease called dengue hemorrhagic fever—defined by severe plasma leakage, bleeding, and multiorgan failure (*10*, *11*). Failure to manage the symptoms of severe dengue can result in death. Yearly, an estimated number of 10,000 deaths result from DENV infections (*3*).

Vaccine development for DENV has proven difficult because of the need for inducing immunization against all four serotypes. To date, there are two live-attenuated tetravalent DENV vaccines available/with market approval. Dengvaxia (Sanofi Pasteur) is licensed in 20 countries, but its use is restricted to people living in endemic areas with a confirmed prior DENV infection (*12*). On the other hand, Qdenga (Takeda) is approved in several countries in Europe, Asia, and Latin America and has been recently prequalified by the World Health Organization (*13*–*17*). Positive efficacy results, regardless of baseline serostatus, have been reported in the Efficacy, Safety and Immunogenicity of Takeda’s Tetravalent Dengue Vaccine (TDV) in Healthy Children (TIDES) study, which was carried out in eight countries in Latin America and Asia (ClinicalTrials.gov, NCT02747927) (*18*, *19*).

There is currently no specific treatment for dengue fever; only supportive care can be given to DENV-infected patients. No antiviral therapies are available for any mosquito-borne virus. Great efforts to find an antiviral agent suitable for the treatment or prevention of dengue have been executed (*20*). One small molecule with promising antiviral activity against DENV is JNJ-A07. This highly potent pan-serotype inhibitor has shown great efficacy in vitro and in mouse infection models against all four DENV serotypes (*21*). JNJ-A07 inhibits the interaction between nonstructural protein 4B (NS4B), a multitransmembrane viral protein in the endoplasmic reticulum, and NS3, another viral protein that contains both a serine protease and a helicase domain (*21*). Furthermore, a molecule in the same chemical space as JNJ-A07, namely JNJ-1802, is currently in clinical development for the prevention and treatment of DENV infections. JNJ-1802 has potent anti-DENV efficacy in nonhuman primates and is safe and well tolerated in humans, with an excellent pharmacokinetic profile (*22*, *23*).

At the moment, prevention and control of dengue infections rely mostly on vector control measures to reduce the population of mosquitoes that are competent to transmit DENV. Nevertheless, some of these control measures also bring major drawbacks (e.g., insecticidal treatments can lead to the emergence of insecticide resistance), or their impact on the mosquito population and virus transmission is not conclusive (e.g., environmental methods that reduce potential mosquito habitats) (*24*–*26*). Therefore, previously unidentified strategies are needed to complement current methods and fight dengue and other mosquito-borne diseases. In such a quest, the concept of using antiviral agents to inhibit virus infection in the mosquito vector is appealing. Adult mosquitoes acquire arboviruses when blood-feeding on infected hosts. Once the virus infection has been established in the mosquito midgut, the virus spreads out to the body cavity and reaches the secondary organs, such as the salivary glands. The virus is next transmitted in the saliva to a naïve host during blood-feeding. In addition, mosquitoes can get exposed to an antiviral drug when they take a blood meal on a human who is being treated with the drug or when they rest on drug-treated surfaces (*27*). Several studies have reported on the effect of the antiparasitic drug ivermectin when ingested during blood-feeding on the mosquito fitness or on *Plasmodium* infection in the mosquito (*28*, *29*). Exposure of *Ae. aegypti* to atovaquone-treated surfaces prevented transmission of chikungunya (CHIKV) virus by the drug-exposed mosquitoes (*30*). Furthermore, six Food and Drug Administration (FDA)–approved drugs with antiflavivirus activity delivered in the blood meal significantly inhibited infection with Zika virus, another flavivirus related to DENV, in mosquito midguts (*31*). In case the antiviral agent taken up by the mosquito is capable of preventing—or interfering with—a DENV infection in the mosquito, virus transmission is likely also stopped. Whether such antiviral strategies involving the mosquito could substantially affect DENV transmission, and thus decrease the number of human infections during an outbreak, remains to be explored.

The main objective of our study was to investigate the antiviral effect of JNJ-A07 on DENV replication and transmission in *Ae. aegypti* mosquitoes. We first confirmed the anti-DENV activity of JNJ-A07 in vitro and ex vivo, using mosquito cells and ex vivo cultured mosquito guts, respectively. Subsequently, we assessed the effect of JNJ-A07 on mosquito survival and fecundity. Next, we determined whether JNJ-A07 could affect DENV replication in mosquitoes when delivered via blood-feeding. We also used our empirical data to define the kinetics of mosquito systemic infections when they ingested JNJ-A07 or the vehicle. In addition, we investigated the clearance time of JNJ-A07 in mosquitoes. Last, a stochastic agent–based model was used to estimate time-dependent individual transmission probabilities and simulate the effect of JNJ-A07 ingestions on DENV outbreaks in silico.

## RESULTS

### JNJ-A07 potently inhibits DENV-2 replication in mosquito cells

The antiviral activity of JNJ-A07 against DENV was assessed in two relevant mosquito cell lines: C6/36 (*Ae. albopictus* derived) and Aag2-AF5 (*Ae. aegypti* derived, AF5 clone) cells. Because DENV serotype 2 (Bangkok strain, MK268692.1) does not induce a cytopathic effect in these mosquito cell lines, the antiviral activity of JNJ-A07 was evaluated by the reduction in intracellular viral RNA, as quantified by quantitative reverse transcription polymerase chain reaction (qRT-PCR).

**Fig. 1.**
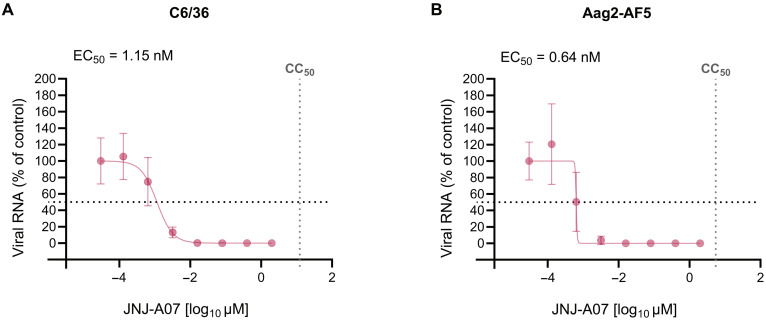
In vitro activity of JNJ-A07 against DENV-2 and cell toxicity in mosquito cell lines. Intracellular viral RNA compared to the virus control (normalized and depicted as percentage of control) was measured by qRT-PCR to evaluate the antiviral activity of JNJ-A07 against DENV-2 in *Ae. albopictus* (C6/36) (**A**) and in *Ae. aegypti* (Aag2-AF5) (**B**) derived cells. The effect of JNJ-A07 on the viability of C6/36 and Aag2-AF5 cells was measured by 3-(4,5-dimethylthiazol-2-yl)-5-(3-carboxymethoxyphenyl)-2-(4-sulfophenyl)-2H-tetrazolium (MTS) assay. The CC_50_ value is indicated by a dotted line perpendicular to the *x* axis. The EC_50_ and CC_50_ were calculated by fitting a nonlinear regression on the empirical data corresponding to two (antiviral activity) and four (toxicity) independent replicates, each with three biological replicates per concentration. Each dot represents the median, and the error bars correspond to the 95% confidence interval.

At the highest concentration tested (2 μM), JNJ-A07 decreased the intracellular viral RNA levels by 6.71 log_10_ and 5.79 log_10_ compared to the virus control in C6/36 and Aag2-AF5 cells, respectively (fig. S1). Lower doses resulted in a descending viral inhibition, indicating a dose-response relationship (with the exception of one of six biological replicates for JNJ-A07 at 0.4 μM in the C6/36 cells). The median effective concentration (EC_50_) value was 1.15 and 0.64 nM for C6/36 and Aag2-AF5 cells, respectively. As for the cytotoxic effects of JNJ-A07 in mosquito cells, the 50% cytotoxic concentration (CC_50_) value was 12.27 and 5.60 μM for C6/36 and Aag2-AF5 cells, respectively (Fig. 1).

### DENV-2 infection is reduced by JNJ-A07 in ex vivo mosquito guts

**Fig. 2.**
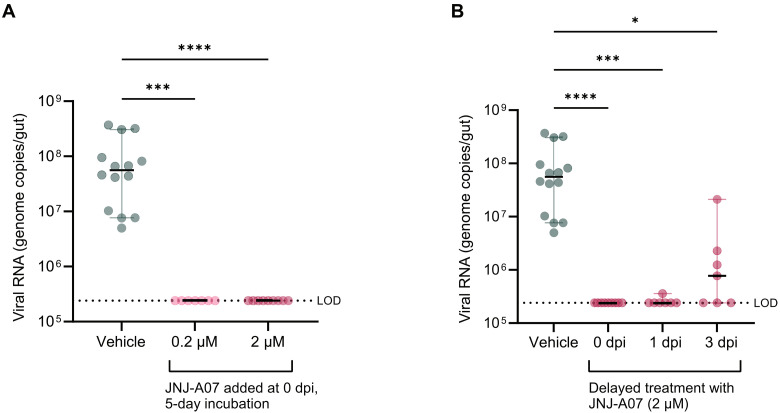
Antiviral activity of JNJ-A07 in ex vivo cultured guts of *Ae. aegypti*. (**A**) DENV RNA levels in guts when incubated in the presence of JNJ-A07 for 5 days, measured at day 5 pi by qRT-PCR. Vehicle, virus control in presence of DMSO (untreated); dpi, days post infection. DMSO concentration was kept at 0.005% in both control and compound conditions. Statistical significance was evaluated with a Kruskal-Wallis test (*P* < 0.0001) and Dunn’s multiple comparison test (vehicle versus 0.2 μM, ***P = 0.0001; vehicle versus 2 μM, ****P < 0.0001). (**B**) DENV RNA levels in guts exposed to JNJ-A07 (2 μM) starting at 0 (time of infection), 1, and 3 days pi, measured at day 5 pi by qRT-PCR. Statistical significance was evaluated with a Kruskal-Wallis test (*P* < 0.0001) and Dunn’s multiple comparison test (vehicle versus 0 dpi, ****P < 0.0001; vehicle versus 1 dpi, ***P = 0.0001; vehicle versus 3 dpi, *P = 0.0145). Each dot represents a mosquito gut. Black lines represent the median, and the colored lines correspond to the 95% confidence interval per condition. Dotted lines depict the limit of detection (LOD) of the assay. Data shown correspond to two independent experiments.

Next, the antiviral activity of JNJ-A07 against DENV infection was examined in ex vivo cultured guts from *Ae. aegypti* mosquitoes to infer potential doses to be used in live mosquitoes. In this model, DENV RNA was undetectable at day 5 post infection (pi) when incubated with JNJ-A07 (0.2 and 2 μM) compared to the untreated infected guts (Fig. 2A).

In addition, DENV-infected ex vivo guts were exposed to JNJ-A07 (2 μM) starting at 0, 1, and 3 days post infection (dpi), and their viral RNA levels were quantified at 5 dpi. Infected guts treated with JNJ-A07 at the moment of infection (0 dpi) or at 1 dpi showed mostly no detectable DENV RNA, except for one gut belonging to the latter group. In contrast, 50% of the guts treated at 3 dpi were DENV infected, albeit the viral RNA levels were significantly lower compared to the control group (mean reduction of ~1.2 log_10_) (Fig. 2B). In addition, JNJ-A07 did not exert a toxic effect on the ex vivo cultured guts, as shown by adenosine triphosphate (ATP) measurements of treated guts in the absence of infection (fig. S2).

### The survival and fecundity of mosquitoes exposed to JNJ-A07 is not significantly altered

**Fig. 3.**
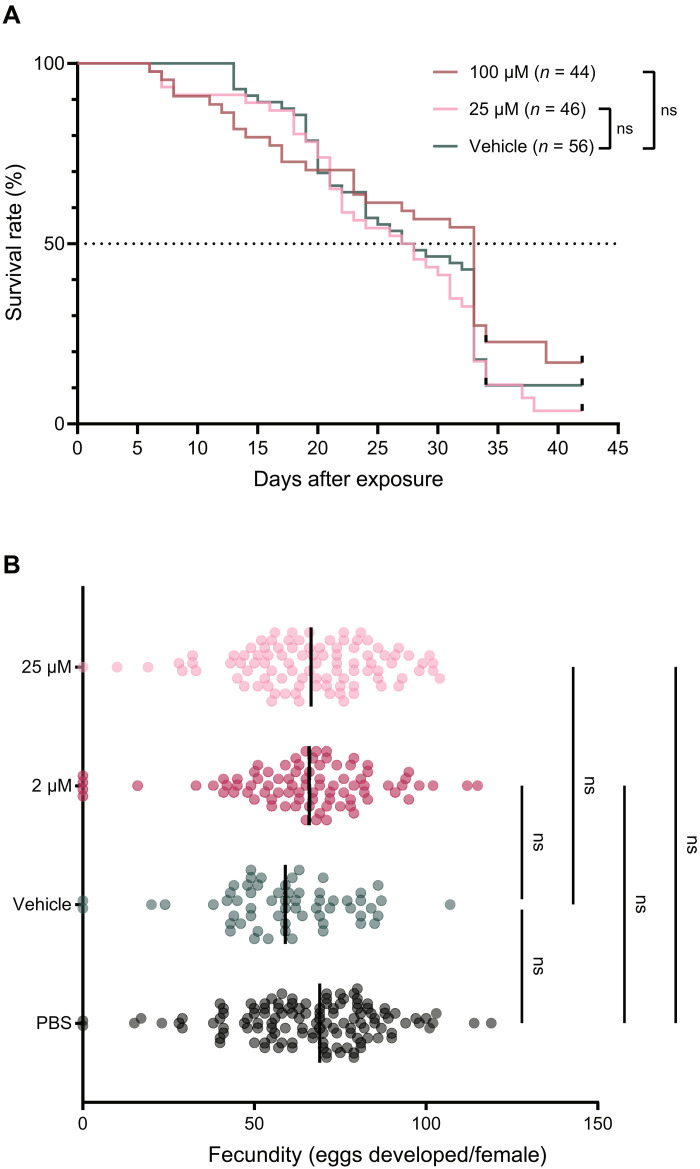
Effect of JNJ-A07 on survival and fecundity of *Ae. aegypti* mosquitoes. (**A**) Mosquito longevity after feeding on a blood meal with JNJ-A07 at 25 and 100 μM, and the vehicle (DMSO, control group) is depicted as percentage of survival. The blood meal for all conditions contained a final concentration of 1% DMSO. Mosquito sample size is indicated per condition by *n*. Statistical significance was assessed by two-sided log-rank (Mantel-Cox) test, *P* = 0.2034. ns, not significant. (**B**) Number of eggs developed in the ovaries of mosquitoes that fed on a blood meal with JNJ-A07 at 2 (*n* = 85) and 25 μM (*n* = 96), and the vehicle (*n* = 61) and PBS (*n* = 122) as control groups. The blood meal for all conditions contained a final concentration of 0.5% DMSO. Each dot represents data from one mosquito, and the lines indicate the median. Statistical significance was assessed with a Kruskal-Wallis test (*P* = 0.0622) and Dunn’s multiple comparison test (vehicle versus PBS, *P* = 0.0610; vehicle versus 2 μM, *P* = 0.2738; vehicle versus 25 μM, *P* = 0.0864; PBS versus 2 μM, *P* > 0.9999; PBS versus 25 μM, *P* > 0.9999). Data shown correspond to two (survival) and three (fecundity) independent experiments.

Mosquitoes were offered a single dose of JNJ-A07 (25 and 100 μM) via a blood meal and were compared to the control group feeding on a blood meal containing dimethyl sulfoxide (DMSO) (vehicle control) alone. Following the blood-feeding, the lifespan of the mosquitoes was controlled daily. The median survival of the vehicle-fed mosquitoes was 27.5 days, while the median survival for the JNJ-A07–fed mosquitoes was 27.5 and 33 days for the 25 and 100 μM dose groups, respectively. Overall, the survival curves between the vehicle- and JNJ-A07–fed mosquitoes were not significantly different, showing that JNJ-A07 had no effect on mosquito longevity even when ingested at very high doses (100 μM) (Fig. 3A).

To study the impact on fecundity, we provided mosquitoes with a single dose of JNJ-A07 (2 and 25 μM) via a blood meal and compared them to the control groups feeding on a blood meal containing phosphate-buffered saline (PBS, mock) or the vehicle alone. At day 4 after the blood meal, the mean number of eggs (± SD) developed per female mosquito was 59 ± 19 for the vehicle control group and 64 ± 22 and 66 ± 20 for the 2 and 25 μM dose groups, respectively. In addition, mosquitoes fed with a mock blood meal (containing PBS only; no DMSO or compound) developed a mean of 66 ± 21 eggs. Thus, JNJ-A07 did not significantly affect egg development when compared to both vehicle- and PBS-fed groups (Fig. 3B).

### JNJ-A07 is a highly effective DENV inhibitor in *Ae. aegypti* mosquitoes

To confirm the antiviral activity of JNJ-A07 in a mosquito infection model, we infected mosquitoes via an artificial blood meal containing DENV-2 in the presence of JNJ-A07 or the vehicle alone. The tested concentrations of JNJ-A07 in the blood meal ranged from 0.005 to 25 μM, and this selection was based on available pharmacokinetic data in DENV-infected mice (*21*).

**Fig. 4.**
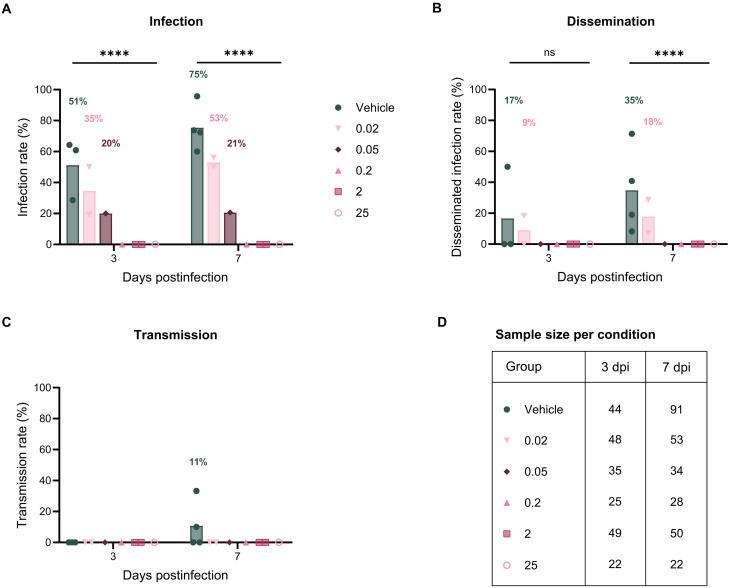
Dose-response effect of JNJ-A07 on the vector competence of *Ae. aegypti* mosquitoes for DENV-2. DENV infection (**A**), disseminated infection (**B**), and transmission (**C**) rates for mosquitoes that fed on a DENV-infectious blood meal spiked with JNJ-A07 at 0.02, 0.05, 0.2, 2, and 25 μM, and the vehicle (control group) were calculated on the basis of the detection of infectious virus at days 3 and 7 pi by plaque assay or focus-forming assay. Sample size across all blood-feeding experiments is depicted for each condition and time point in (**D**). Each symbol represents an independent blood-feeding experiment where said condition was tested, and the bar height corresponds to the mean rate. Percentages appear above the bar when it is different than 0%. Statistical significance was evaluated with a Fisher’s exact test (IRs at 3 and 7 dpi, ****P < 0.0001; DIRs at 3 dpi, *P* = 0.0677, and 7 dpi, ****P < 0.0001). ns, not significant.

At 25, 2, and 0.2 μM, JNJ-A07 exposure via the blood meal at the time of infection resulted in a complete inhibition of DENV infection in *Ae. aegypti* mosquitoes. While the vehicle control group had infection rates (IR) of 51 and 75% at days 3 and 7 pi, respectively, there were no DENV-infected mosquitoes detected at these time points for the JNJ-A07–exposed groups. Disseminated infection rates (DIR) in the vehicle control group—indicative of virus spread to the secondary organs—were 17 and 35% at days 3 and 7 pi, respectively. A transmission rate (TR) of 11% was observed for the vehicle control group at day 7 pi (Fig. 4, A to C).

In contrast, lower doses of JNJ-A07 (0.05 and 0.02 μM) in the blood meal resulted in DENV infections in *Ae. aegypti* mosquitoes, albeit to substantially smaller proportions of infected mosquito bodies when compared to the vehicle control group. The IR for 0.05 μM–exposed mosquitoes was 20 and 21% at days 3 and 7 pi, respectively. The IR for 0.02 μM–exposed mosquitoes was slightly higher, with 35 and 53% at days 3 and 7 pi, respectively. Dissemination to secondary organs was not observed in the 0.05 μM–exposed mosquitoes at days 3 and 7 pi, and consequently also no virus transmission was recorded. The DIR was 9 and 18% for 0.02 μM–exposed mosquitoes at days 3 and 7 pi, respectively, but no virus in the mosquito saliva was detected (Fig. 4, A to C).

Last, concentrations lower than 0.02 μM—0.01 and 0.005 μM—showed infection kinetics similar to the vehicle control group when assessed for infectious virus at day 7 pi. The IRs for 0.01 or 0.005 μM–exposed groups at day 7 pi were 61 and 76%, respectively. No significant difference was observed between the IRs of the vehicle- and 0.01 or 0.005 μM–exposed mosquitoes (fig. S3).

### JNJ-A07 efficiently blocks DENV infection in mosquitoes in a prophylactic setting, while disrupting an ongoing viral infection when administered in a therapeutic setting

**Fig. 5.**
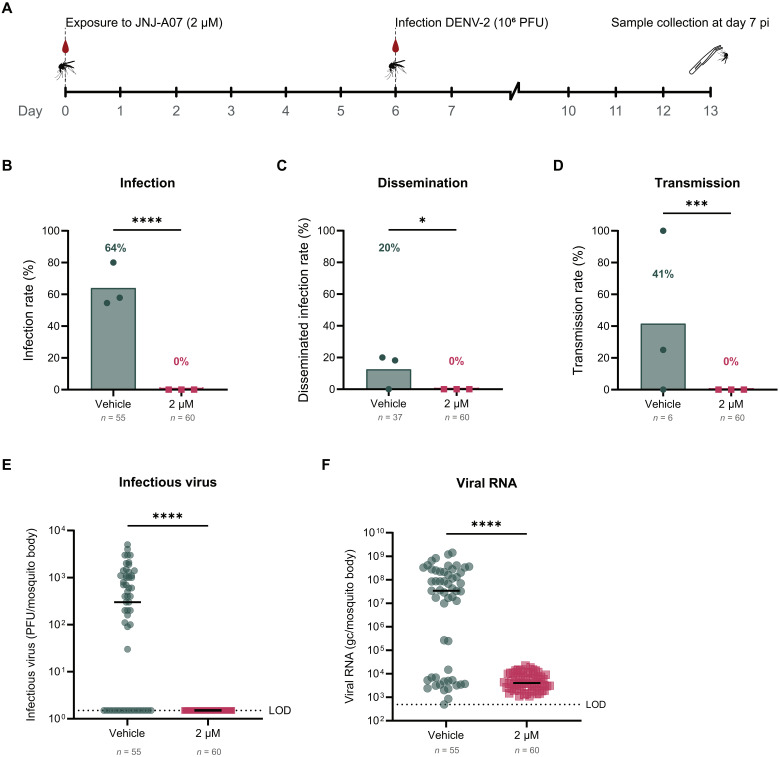
Efficacy of JNJ-A07 on DENV-2 infection in mosquitoes when ingested via the blood meal in a prophylactic setting. (**A**) Timeline depicting the design of the experiment, in which mosquitoes received a blood meal spiked with JNJ-A07 at 2 μM or the vehicle (control group), and 6 days later, they received a second blood meal containing DENV-2. Mosquitoes were euthanized at day 7 pi (i.e., 13 days after exposure). Infectious virus was detected by plaque assay. DENV infection (**B**), disseminated infection (**C**), and transmission (**D**) rates for mosquitoes that were exposed to JNJ-A07 before the infectious blood meal. Each dot or square represents an independent blood-feeding experiment in (B) to (D), and the bar height corresponds to the mean. Mosquito sample size is indicated per condition by *n*. Percentages appear above the bar. Statistical significance was evaluated with a Fisher’s exact test (IRs, ****P < 0.0001; DIRs, *P = 0.0191; TRs, ***P = 0.0004). Infectious virus (**E**) and DENV RNA levels (**F**) in the mosquito bodies when pre-exposed to the vehicle and 2 μM of JNJ-A07, measured by plaque assay and qRT-PCR, respectively. Each circle or square represents a biological replicate (one mosquito) in (E) and (F), and the black lines correspond to the median. The dotted line represents the LOD of the assay. Mosquito sample size is indicated per condition by *n*. Statistical significance in (E) and (F) was assessed with a Mann-Whitney test (****P < 0.0001). Data shown correspond to three independent blood-feeding experiments. gc, genome copies.

The antiviral effect of an in vivo relevant concentration (2 μM) of JNJ-A07 was next assessed in a more realistic scenario, in which the virus and the drug were delivered via consecutive blood meals to the mosquitoes. First, we evaluated a pre-exposure prophylaxis scenario by providing a JNJ-A07–spiked blood meal followed by a DENV-infectious blood meal 6 days later (Fig. 5A). At 7 dpi (13 days after exposure to JNJ-A07), 64% of the vehicle–pre-exposed mosquitoes had an established DENV infection. In contrast, none of the mosquitoes that received JNJ-A07 before the DENV-infectious blood meal had detectable infectious virus titers, and consequently also no dissemination nor transmission was observed (Fig. 5, B to E). Furthermore, pre-exposure to JNJ-A07 induced a 4.4 log_10_ reduction in viral genome copies per body compared to the vehicle-exposed group (mean of 6.3 × 10^3^ genome copies per body versus 1.6 × 10^8^ genome copies per body, respectively) (Fig. 5F).

**Fig. 6.**
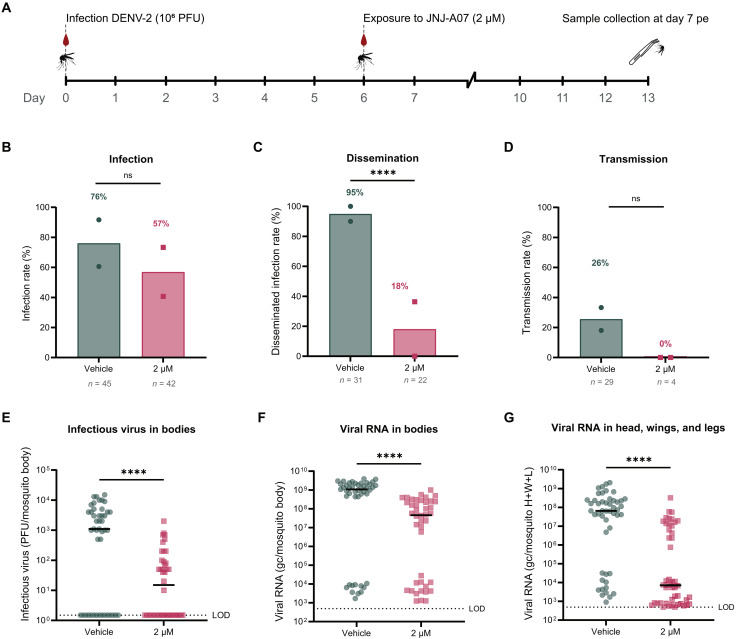
Efficacy of JNJ-A07 on DENV-2 kinetics when ingested via the blood meal in a therapeutic setting. (**A**) Timeline depicts the design of the experiment, in which mosquitoes received a blood meal containing DENV-2, and 6 days later, they received a second blood meal spiked with JNJ-A07 at 2 μM or the vehicle (control group). Mosquitoes were euthanized at day 7 post-exposure (pe) (i.e., 13 dpi). Infectious virus was detected by plaque assay. DENV infection (**B**), disseminated infection (**C**), and transmission (**D**) rates for mosquitoes that were exposed to JNJ-A07 after the infectious blood meal. Each dot or square represents an independent blood-feeding experiment in (B) to (D), and the bar height corresponds to the mean. Mosquito sample size is indicated per condition by *n*. Percentages appear above the bar. Statistical significance was evaluated with a Fisher’s exact test (IRs, *P* = 0.1295; DIRs, ****P < 0.0001; TRs, *P* = 0.5503). ns, not significant. Infectious virus (**E**) and DENV RNA levels (**F**) in the bodies of mosquitoes exposed after infection to the vehicle and JNJ-A07 measured by plaque assay and qRT-PCR, respectively. (**G**) DENV RNA levels in the heads, wings, and legs of mosquitoes exposed after infection to the vehicle and JNJ-A07 measured by qRT-PCR. Each circle or square represents a biological replicate (one mosquito) in (E) to (G), and the black lines correspond to the median. The dotted line represents the LOD of the assay. Mosquito sample size is indicated per condition by *n*. Statistical significance in (E) to (G) was assessed with a Mann-Whitney test (****P < 0.0001). Data shown correspond to two independent experiments. H+W+L, head, wings, and legs. LOD, limit of detection of the corresponding assay.

Secondly, mosquitoes were provided with a DENV-infectious blood meal 6 days before feeding on a JNJ-A07–spiked blood meal (2 μM), resembling a post-exposure scenario (Fig. 6A). Mosquitoes exposed to the vehicle 6 days after the infectious blood meal had an IR of 76% at 13 dpi, whereas the JNJ-A07–exposed mosquitoes had a lower IR (57%), although not statistically significant. On the contrary, the DIR of the JNJ-A07–exposed group was significantly lower (18%) compared to the vehicle control group (95%). The TR of the vehicle control group was 26% at 13 dpi, while no virus transmission was observed in the JNJ-A07–exposed mosquitoes (Fig. 6, B to D). Moreover, the mean titer of infectious virus in the vehicle-exposed infected mosquitoes was 4.6 × 10^3^ plaque-forming units (PFU) per mosquito body, significantly higher (an increase of 1.2 log_10_) than the mean titer in the JNJ-A07–exposed infected mosquitoes (2.9 × 10^2^ PFU per body) (Fig. 6E). In addition, viral RNA levels were also significantly decreased in the bodies (by 0.9 log_10_) and head, wings, and legs (by 1.3 log_10_) of the JNJ-A07–exposed group compared to the vehicle control group (Fig. 6, F and G).

### JNJ-A07 persists in the mosquito body for 7 days after ingestion via the blood meal

**Fig. 7.**
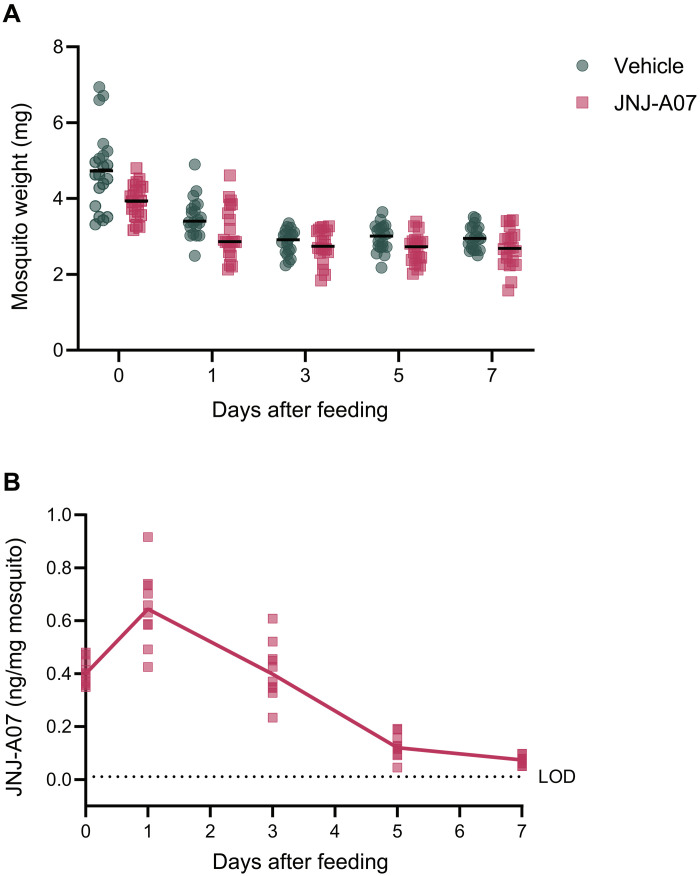
Bodyweight– and concentration-time course of JNJ-A07 in *Ae. aegypti* mosquitoes when delivered via the blood meal. (**A**) The bodyweight of each mosquito that fed on a blood meal spiked with the vehicle (control group) or JNJ-A07 at 2 μM was monitored for 7 days after feeding. The black lines represent the median. Each dot or square represents a single mosquito (JNJ-A07, *n* = 100; vehicle, *n* = 100). (**B**) The concentration of JNJ-A07 was measured in 10 mosquitoes per time point for 7 days after feeding by liquid chromatography–mass spectrometry. The line connects the medians for each time point. The dotted line represents the limit of quantification of the assay. Data shown correspond to one blood-feeding experiment.

We further analyzed the uptake and clearance of JNJ-A07 in the mosquitoes following a blood meal containing 2 μM of JNJ-A07. The mean weight of females measured directly after the feeding was 3.9 mg. During the first 24 hours after feeding, the mosquitoes lost a significant amount of weight, as expected. No further changes were observed in the bodyweight when measured at later time points (Fig. 7A).

Right after the blood-feeding, the concentration of JNJ-A07 in the fed mosquitoes’ bodies was on average 0.41 ± 0.05 (SD) ng/mg of mosquito. In the next 24 hours, the concentration of JNJ-A07 in the mosquitoes increased to an average of 0.65 ± 0.14 ng/mg of mosquito, most likely as a result of bodyweight loss due to liquid excretion by the mosquito after the blood meal. Over the next 6 days, 89% of the ingested JNJ-A07 was cleared. JNJ-A07 was still detected at day 7 after feeding at an average concentration of 0.07 ± 0.02 ng/mg of mosquito (Fig. 7B), which would correspond to an average of 1 ng/ml of sample.

### JNJ-A07 exposure to mosquitoes could potentially affect the proportion of dengue outbreaks in humans

**Fig. 8.**
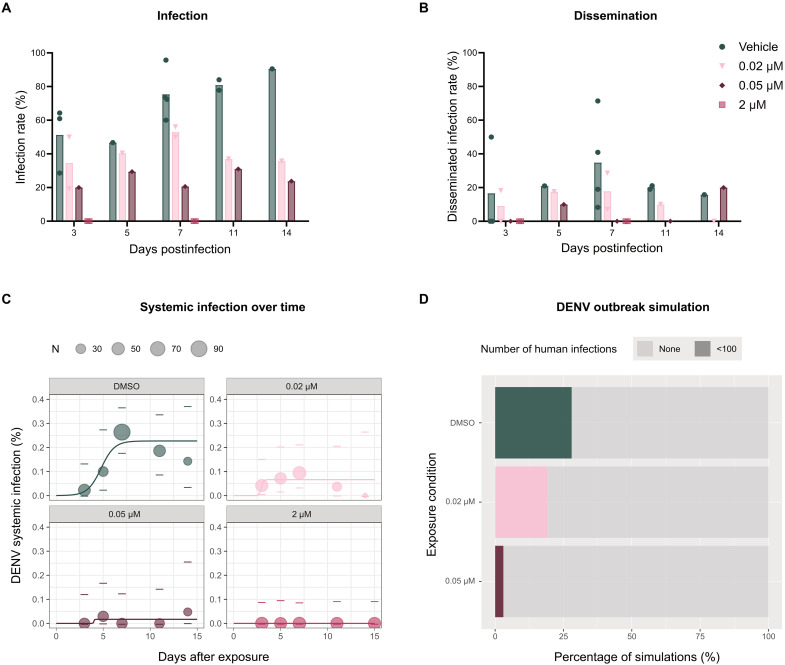
Modeled effect of the reductions in DENV systemic infection, as a result of JNJ-A07 exposure, on the magnitude of DENV outbreaks. DENV infection (**A**) and disseminated infection (**B**) rates for mosquitoes that fed on a DENV-infectious blood meal spiked with JNJ-A07 at 0.02, 0.05, and 2 μM, and the vehicle (control group). Infectious virus was detected at days 3, 5, 7, 11, and 14 pi by plaque assay or focus-forming assay. Each symbol represents an independent blood-feeding experiment. Data for the 2 μM dose were only collected for days 3 and 7 pi; data for days 5, 10, and 14 were extrapolated from available time points. The bar height corresponds to the mean. (**C**) The cumulative prevalence of systemic infection over time after exposure (and after infection) is displayed for each condition. A logistic model was fitted to all exposure conditions based on three parameters. For each condition, dots represent the empirical data. The size of the dots represents the sample size. The dashes correspond to the 95% confidence interval of the prevalence. The colored line represents the logistic fit of the dataset. (**D**) Proportion of simulations that resulted in ≥100, <100, or no secondary human infections using the *K*, *B*, and *M* values empirically measured. Statistical significance was assessed by the test of Equal or Given proportions (0.02 μM versus 0.05 μM, *P = 0.001; 0.02 μM versus vehicle, *P* = 0.182; 0.05 μM versus vehicle, *P = 0).

A logistic model was fitted to the empirical data to model the probabilities of systemic DENV infection in *Ae. aegypti* mosquitoes over time (Fig. 8, A and B, and Table 1). Four parameters that described the kinetics of viral infection in the mosquitoes were estimated (*K*, *B*, Δ*t*, and *M*) for each condition, as shown in Table 2. Notably, for the condition of JNJ-A07 at 2 μM, data were available for only two time points (3 and 7 dpi); however, it was possible to extrapolate the prevalence of systemic infection in silico to perform the following analysis based on available data. The cumulative prevalence of systemic infection for the vehicle condition showed the highest prevalence of infection (26%) at day 7 pi after exposure, while the prevalence was null for the mosquitoes exposed to 2 μM of JNJ-A07 (Fig. 8C). For the vehicle control group, the saturation level *K* was 22%, and the Δ*t* (time required to rise from 10 to 90% of the saturation level) was 3.13 days. In the groups exposed to JNJ-A07, the saturation *K* level was 6 and 1% for 0.02 and 0.05 μM, respectively, whereas the Δ*t* was 0.47 and 0.14 days for the respective concentrations. This indicates that the JNJ-A07 concentrations used in the blood meal effectively disrupted viral dissemination to the mosquito secondary organs, when compared to the vehicle control group.

**Table 1.** IR, DIR, and TR of *Ae. aegypti* mosquitoes in each treatment group. The letter following the SD refers to the number of independent replicates; a = 1, b = 2, c = 3, d = 4. NA, not applicable.

Days pi	Vehicle			
	No.	IR% (SD)	DIR% (SD)	TR% (SD)
3	44	51 (20)^c^	17 (29)^c^	0 (0)^c^
5	30	47^a^	21^a^	0^a^
7	91	75 (15)^d^	35 (28)^d^	11 (16)^d^
11	43	81 (4.4)^b^	20 (1.4)^b^	0 (0)^b^
14	21	90^a^	16^a^	0^a^
	**JNJ-A07 0.02 μM**			
3	48	35 (22)^b^	9 (13)^b^	0 (0)^b^
5	42	40^a^	18^a^	0^a^
7	53	53 (4.2)^b^	18 (15)^b^	0 (0)^b^
11	27	37^a^	10^a^	0^a^
14	14	36^a^	0^a^	0^a^
	**JNJ-A07 0.05 μM**			
3	35	20^a^	0^a^	0^a^
5	34	29^a^	10^a^	0^a^
7	34	21^a^	0^a^	0^a^
11	29	31^a^	0^a^	0^a^
14	21	24^a^	20^a^	0^a^
	**JNJ-A07 2 μM**			
3	49	0 (0)^b^	0 (0)^b^	0 (0)^b^
5	NA	NA	NA	NA
7	50	0 (0)^b^	0 (0)^b^	0 (0)^b^
11	NA	NA	NA	NA
14	NA	NA	NA	NA

**Table 2.** Parameter estimates of DENV systemic infection kinetics for all exposure conditions.

Condition	*K* (%)	*B* (% day^−1^)	Δ*t* (days)	*M* (days)
Vehicle	22.70	1.40	3.13	4.78
0.02 μM	6.62	9.25	0.47	2.94
0.05 μM	1.69	30.50	0.14	4.00
2 μM	0	0.96	4.56	1.93

An agent-based model was further applied to estimate the epidemiological significance of the differences observed in systemic DENV infection across all conditions tested, in a simulated environment (Fig. 8D). None of the conditions (vehicle, 0.02 μM, or 0.05 μM exposed) displayed a large-scale outbreak (≥100 infected humans) in this model. The proportion of small-scale outbreaks (<100 infected humans) was 28% for the vehicle control group, while the mean proportion of humans infected in the 0.02 μM– and the 0.05 μM–exposed conditions were 19 and 3%, respectively. The *K*, *B*, and *M* values were unstable for the dose of 2 μM given the zero infection prevalence at all data points; hence, the outbreak simulation algorithm could not be performed for this group. In addition, the proportion of small-scale outbreaks was significantly different for the vehicle- versus the 0.05 μM–exposed groups but not for the vehicle-versus the 0.02 μM–exposed groups. This further validates the potential of JNJ-A07 at concentrations above 0.05 μM in the blood meal to significantly and effectively reduce transmission and, consequently, lower the number of small-scale DENV outbreaks in the simulated population.

## DISCUSSION

DENV remains a significant global health threat, causing millions of infections every year. The primary vector responsible for DENV transmission is the *Ae. aegypti* mosquito. Despite extensive efforts to control mosquito populations and reduce disease burden, there is a strong need for new effective strategies. While there is no antiviral treatment for DENV infections yet, great progress has been achieved in the search for antiviral drugs. We here explored a promising avenue involving the mosquito vector: the ingestion of antiviral drugs by mosquitoes during their blood-feeding on drug-treated patients. To study the antiviral effect of drug ingestion by mosquitoes, we have used JNJ-A07, a molecule belonging to the same chemical space as JNJ-1802 (*21*), in our experimental setup as a surrogate for the clinical candidate.

Considering that the mosquito vector has an important role in the transmission of DENV, and any mosquito-borne virus for that matter, the importance of evaluating potential inhibitors in mosquito-derived models becomes evident. If an inhibitor can disrupt the arbovirus infection cycle within the mosquito body, then virus dissemination will likely be affected, potentially reducing virus transmission from an infected mosquito to a naïve human. Here, we confirmed the potent antiviral activity of JNJ-A07 against DENV serotype 2 (isolate DENV-2/TH/1974) in two mosquito cell lines derived from *Ae. albopictus* and *Ae. aegypti*. The EC_50_ values in both mosquito cell lines were comparable to and within the range of activity observed for JNJ-A07 against other DENV-2 strains in C6/36 cells (*21*). Furthermore, the inhibitory effect was retained in *Ae. aegypti* guts cultured ex vivo in the presence of JNJ-A07. A therapeutic setting was simulated by adding JNJ-A07 at different time points after DENV infection in the ex vivo guts. Delayed treatment of the mosquito guts resulted in progressively more guts becoming DENV infected; however, the viral RNA loads in treated guts were still significantly lower compared to the control group at all tested time points. Such findings align with the mechanism of action of JNJ-A07: It prevents the interaction between the viral proteins NS3 and NS4B, hindering the formation of complexes crucial for viral replication, without disrupting the already formed NS3-NS4B complexes (*21*, *32*).

Previously published studies have investigated the antiviral activity of several molecules against a variety of arboviruses in mosquito cell lines, in addition to mammalian cell lines [reviewed in (*27*)]. However, very few molecules have been assessed for their antiviral efficacy against arboviruses in live mosquito infection models. The broad-spectrum antiviral drug favipiravir was proven efficient against CHIKV infection in Vero cells, but not in C6/36 or Aag2-AF5 cells, or when provided to *Ae. aegypti* mosquitoes in a CHIKV-infectious blood meal (*33*). An extensive screen of 55 FDA-approved antiviral drugs against flaviviruses yielded two drugs with mosquitocidal activity and six drugs that significantly decreased ZIKV infection in *Ae. aegypti* mosquito midguts (*31*). In addition, ingestion of bafilomycin or mycophenolic acid via a DENV-infectious blood meal by *Ae. aegypti* mosquitoes significantly reduced DENV titers in mosquito bodies (*34*). Although only infection in the midgut was evaluated in these studies and not dissemination nor transmission, these results hint at the potential for an antiviral drug ingested by mosquitoes to influence arbovirus replication. In this context, our study has demonstrated that JNJ-A07 significantly reduced DENV infection, dissemination, and transmission when ingested via the infectious blood meal at preclinically relevant concentrations. The concentrations of JNJ-A07 added in the blood meal of live mosquitoes were selected on the basis of previously published studies with JNJ-A07 in mice. In a DENV mouse model, a twice-daily oral dose of JNJ-A07 at 3 or 10 mg/kg reduced the viral RNA with 1.9 or 3.6 log_10_ compared to the control group at 3 dpi. Furthermore, pharmacokinetics in the mouse model showed that these oral doses of JNJ-A07 yielded a maximum plasma concentration of 1.44 or 3.60 μM, respectively (*21*). Considering that mosquitoes feed on whole blood and not on plasma, the abovementioned concentrations have been corrected to 0.92 or 2.31 μM for the 3 or 10 mg/kg doses, respectively. Therefore, a dose of 2 μM was deemed as suitable for studies in live mosquitoes. This preclinically relevant concentration of 2 μM completely prevented DENV infection in mosquitoes.

As the therapeutic window for an arbovirus infection is rather short, promising antiarboviral drugs could be provided as a prophylaxis (*22*). Hence, this drug could be valuable for travelers to or populations in dengue-endemic regions, not only to prevent DENV infection in treated people but also to potentially decrease DENV transmission from humans to naïve mosquitoes. We have investigated a potential additional layer of protection inherent in this strategy, in which naïve mosquitoes would ingest a potent DENV inhibitor via the blood meal when feeding on a treated human. We simulated such a prophylactic setting in live mosquitoes in which JNJ-A07 was extremely effective: It completely blocked DENV replication in the mosquitoes, illustrated by the lack of infectious virus in the bodies of pre-exposed mosquitoes. This inhibitory effect could be explained by the long-term presence of JNJ-A07 in the mosquito bodies, even after 6 days of ingesting JNJ-A07 via the blood meal. We hypothesize that JNJ-A07 can traverse the peritrophic matrix in the gut and be absorbed by the midgut cells, as the levels of JNJ-A07 at day 3 after feeding (after the digestion of the blood meal has passed) are comparable to the values for day 0 after feeding (at which mosquitoes had just taken the blood meal). Furthermore, when DENV-infected mosquitoes were exposed to JNJ-A07 6 days after the infection, a notable reduction was observed in the proportion of mosquitoes with a disseminated DENV infection compared to the vehicle control group (vehicle, 95% versus JNJ-A07, 18%). Moreover, a modeling exercise using an agent-based mathematical model showed that the inhibitory effect of JNJ-A07 at 0.05 μM and higher concentrations could potentially lead to a significantly reduced proportion of dengue outbreaks, compared to the vehicle-treated condition.

Viral infection kinetics are known to be influenced by the specific virus-mosquito strain pairing. The Paea strain of *Ae. aegypti* started from mosquitoes collected in French Polynesia and has been reared in the laboratory since 1994. Its high susceptibility for DENV has led to this strain being fairly used in vector competence studies (*35*–*37*). In our hands, this strain of mosquitoes was able to become infected with DENV-2 Bangkok strain as shown in this study (MK268692.1), as opposed to when provided an infectious blood meal containing the DENV-2 Rega Lab strain (MW741553), a laboratory-adapted virus strain. Higher IRs, and possibly dissemination rates, in our vehicle control group might have been achieved if using a higher virus titer in the blood meal, as the proportion of infected mosquitoes is positively associated with the virus ingested in the blood meal (*35*, *38*). However, for the purpose of this research, the virus inoculum used to infect mosquitoes was epidemiologically relevant as it is representative of DENV viremia in humans and falls close to the range of the 50% oral infectious dose (OID_50_)—defined as the virus concentration that results in 50% infected mosquitoes—reported previously for six DENV-2 isolates (10^4.61^ to 10^6.02^ PFU/ml) (*39*, *40*).

Last, another important aspect that needs to be considered is the possibility of drug resistance emergence. Because mosquitoes transmit DENV to and from humans, there is the potential that a drug-resistant DENV is ingested from a treated human by a mosquito and potentially transmitted to another human host. One study using drug-resistant CHIKV viruses reported that one of the tested drug-resistant variants was able to be transmitted efficiently by *Ae. aegypti* (*41*). An antiviral drug with a high barrier to resistance would be desirable to avoid such outcome. For JNJ-A07, fully resistant variants have been reported to emerge after an extensive period of resistance selection in vitro (>40 weeks). Whole-genome sequencing revealed several mutations within the NS4B gene, of which the amino acid changes L94F, T108I, and V91A were present in 100% of the viral population at the end of the selection process (*21*). The fitness of these drug-resistant variants in mosquitoes remains unexplored; however, these variants were reported to be unable to replicate in mosquito cells, which might hint to their inability to thrive in live mosquitoes.

In this study, we have demonstrated that JNJ-A07 is an effective DENV-2 inhibitor in mosquito-derived models (in vitro and ex vivo) as well as in live laboratory-reared *Ae. aegypti* mosquitoes. Notably, both prophylactic and therapeutic exposure to JNJ-A07 also prevented and reduced DENV infection and dissemination in mosquitoes, respectively. According to modeling using an agent-based model, exposure of mosquitoes to JNJ-A07 via the blood meal led to a potentially lower proportion of DENV outbreaks in humans. Further research is needed to confirm this effect for other DENV serotypes. Overall, our findings contribute to characterizing JNJ-A07 as a DENV transmission-blocking agent in mosquitoes and shed light on the potential epidemiological impact that the ingestion of such agent by the mosquitoes could have on DENV outbreaks in humans. Building upon our research, a highly potent anti-DENV drug could not only reduce viral infection in humans but also act as an extra layer of protection by reducing virus transmission from mosquitoes to untreated humans.

## MATERIALS AND METHODS

### Compounds

JNJ-A07 was obtained from Janssen Pharmaceutica (Beerse, Belgium). The compound was dissolved in 100% DMSO as a 1, 4, or 5 mM stock, stored at 4°C.

### Cells

*Ae. albopictus*–derived larval cells [C6/36; American Type Culture Collection (ATCC), CCL-1660] were cultivated in Leibovitz’s L-15 medium, supplemented with 10% fetal bovine serum (FBS), 1% penicillin-streptomycin (Pen-Strep), 1% nonessential amino acids (NEAA), and 1% Hepes buffer. *Ae. aegypti*–derived cells [Aag2-AF5 clone; a gift from K. Maringer; University of Surrey, UK (*42*)] were cultivated in Schneider’s *Drosophila* medium, supplemented with 10% FBS, 1% Pen-Strep, and 2 mM l-glutamine. Both mosquito cell lines were incubated at 28°C in the absence of CO_2_.

Baby hamster kidney (BHK) cells (ATCC, CCL-10) were maintained in Dulbecco’s modified Eagle’s medium, supplemented with 10% FBS, 1% sodium bicarbonate, and 1% l-glutamine. BHK cells were cultured at 37°C and 5% CO_2_. For cell culture assays containing virus or virus-infected material, the concentration of FBS in the medium was reduced to 2%. All the cell culture media and supplements were purchased from Gibco, Thermo Fisher Scientific (Belgium).

### Viruses

DENV serotype 2 (DENV-2/TH/1974, isolated in 1974 from human serum collected in Bangkok, Thailand; GenBank MK268692.1) was provided by A. Failloux (Institut Pasteur, France) (*35*). Virus stocks were prepared by passaging the isolate on C6/36 cells, and viral titers were determined by performing plaque assays on BHK cells, as described further in this section.

### Antiviral assays in mosquito cells

C6/36 and Aag2-AF5 cells were preseeded at a density of 1 × 10^5^ cells per well in 96-well tissue culture plates (BD Falcon, Switzerland) and allowed to adhere overnight. Next, dilutions of JNJ-A07 were prepared in the corresponding medium, and subsequently, the cells were infected with DENV-2 at a multiplicity of infection of 0.01. Following 7 days of incubation at 28°C, without CO_2_, intracellular RNA was extracted using the Cells-to-cDNA cell lysis buffer (Invitrogen, Thermo Fisher Scientific), after which qRT-PCR was performed to determine DENV genome copies. The concentration of JNJ-A07 required to efficiently inhibit virus replication by 50% (EC_50_) was calculated by logarithmic extrapolation.

### Drug toxicity assays in mosquito cells

C6/36 and Aag2-AF5 cells were preseeded at a density of 1 × 10^5^ cells per well in a 96-well tissue culture plate (BD Falcon) and allowed to adhere overnight. Next, twofold dilutions of JNJ-A07 were prepared in the corresponding medium. The cells were incubated in the presence of JNJ-A07 dilutions at 28°C, without CO_2_ for 7 days. After incubation, the colorimetric MTS assay was performed to assess cell viability following the manufacturer’s protocol (Promega, The Netherlands). In brief, the culture medium was removed from the 96-well plate. Next, MTS diluted in culture medium (1:10) was added to the cells and incubated for 1 hour. Then, the absorbance was measured at 490 nm using a Spark Multimode Microplate reader (Tecan Trading AG, Switzerland). The concentration required to reduce the cell viability by 50% (CC_50_) was calculated by logarithmic extrapolation.

### Plaque assay

BHK cells were preseeded at a density of 2.5 × 10^5^ cells per well in a 24-well tissue culture plate (BD Falcon) and allowed to adhere overnight. Next, the confluent monolayers were inoculated with serial 10-fold dilutions of each sample (supernatant) collected. The dilutions were incubated with the cells at 37°C for 2 hours, with 5% CO_2_. Following incubation, the inoculum was removed and replaced with carboxymethyl cellulose (CMC) 0.8% diluted in RPMI 1640 medium. At day 7 pi, 4% paraformaldehyde was added to each well on top of the CMC medium and allowed to fix for 1 hour, after which the overlay mixture was discarded, and the wells were carefully washed with water. The plates were air dried and stained with 1% crystal violet solution (Sigma-Aldrich, USA). Plaques were counted to determine the PFU for each sample.

### qRT-PCR for DENV-2

One-step, qRT-PCR was performed in a final volume of 20 μl, consisting of 6 μl of ribonuclease-free water (Promega, USA), 10 μl of SYBR Green master mix (Bio-Rad, USA), 0.3 μl of each forward D2-F and reverse primer D2-R [to a final concentration of 900 nM for each primer (*43*)], 0.4 μl of reverse transcriptase (Bio-Rad, USA), and 3 μl of test sample. Notably, another primer pair constructed in-house was briefly used at the start of this project (forward: 5′-CAGATCGGA-GCTGGAGTTTAC-3′, reverse: 5′-TTTTGACGTCCGCCCATGAA-3′). The assays were performed using the QuantStudio 5 Real-Time PCR System (Thermo Fisher Scientific) with the following cycling conditions: 30 min at 48°C, 10 min at 95°C, followed by 40 cycles of 15 s at 95°C and 1 min at 60°C. For quantification, standard curves were generated each run using 10-fold serial dilutions of viral RNA isolated from DENV-2/TH/1974 Bangkok virus stocks.

### Rearing of *Ae. aegypti* mosquitoes

*Ae. aegypti* eggs (Paea; Papeete, Tahiti, collected in 1994) were obtained via the Infravec2 consortium and reared in the laboratory (*35*). Eggs were hatched in dechlorinated tap water. After hatching, groups of 400 larvae were transferred into trays containing 3 liters of dechlorinated tap water and fed daily with a yeast tablet (Gayelord Hauser, France) until they reached the pupal stage. Adult mosquitoes were fed with a 10% sugar solution, and their cages were maintained at 28° ± 1°C and 75% relative humidity, with a light/dark cycle of 16/8 hours.

When mosquitoes were intended for use in ex vivo experiments, pupae were placed in plastic containers inside 800-ml cardboard cups for their emergence. Adults were fed with cotton balls soaked in 10% sugar solution supplemented with penicillin (100 U/ml) and streptomycin (100 μg/ml) (Pen-Strep), respectively.

### Antiviral and viability assays in ex vivo cultured mosquito guts

Unfed, antibiotic-treated female mosquitoes (3 to 7 days old) were cold anesthetized, and their guts dissected from their body cavity, as described previously (*44*). The mosquito guts were cultured in a 96-well tissue culture plate containing Leibovitz’s L-15 medium, supplemented with 2% FBS, 1% Pen-Strep, kanamycin (50 μg/ml), and amphotericin B (0.25 μg/ml; Sigma-Aldrich). Culture plates were kept at 28°C, without CO_2_.

The antiviral activity of JNJ-A07 was evaluated in the ex vivo mosquito guts, as detailed in (*44*). In brief, JNJ-A07 dilutions prepared in culture medium were added to the guts in a 96-well plate. Immediately after, guts were infected by adding DENV-2 (1 × 10^5^ PFU/ml). The guts were incubated with JNJ-A07 and virus together for 2 hours at 28°C, without CO_2_. A virus control condition was included, containing guts incubated with virus and the vehicle DMSO (final concentration of 0.005% DMSO) but without JNJ-A07. Following 2 hours of incubation, the inoculum was removed, and the guts were washed twice with medium to remove nonadsorbed virus. Culture medium containing JNJ-A07 was then added to the treated guts, while culture medium (containing the vehicle) was added to the virus control guts. The guts were incubated for 5 days at 28°C, without CO_2_, after which the guts were collected for virus quantification by qRT-PCR and plaque assay.

The toxicity of JNJ-A07 in the ex vivo cultured guts was assessed by an ATP-based assay using Cell Titer-Glo 3D Reagent (Promega, USA) and following the manufacturer’s protocol with few modifications. In brief, dissected mosquito guts were incubated with JNJ-A07 (2 μM) or the vehicle for 7 days (using culture medium without phenol red) in a 96-well plate. Each well contained one mosquito gut. DMSO concentration was kept at 0.05% in both vehicle and compound conditions. At day 7, the culture medium containing JNJ-A07 or the vehicle was removed from each gut-containing well, and 100 μl of fresh culture medium was added. Then, 100 μl of Cell Titer Glo-3D Reagent was added to each test well. Next, the 96-well plate was placed on a shaker at 400 rpm for 5 min to induce cell lysis. The signal was allowed to stabilize by incubating the plate at room temperature, protected from the light, for 25 min. After incubation, 100 μl of the supernatant from each well was transferred to a black plate (Greiner, Austria) with a black sticker added to obscure the bottom of the wells. Last, luminescence was recorded using a Spark Multimode Microplate reader (Tecan Trading AG, Switzerland).

### Mosquito survival and egg development assays

The effect of JNJ-A07 on the mosquito’s life span and fecundity was assessed by exposing the mosquitoes to a blood meal spiked with JNJ-A07 at different concentrations. Female mosquitoes (5 to 10 days old) were gathered in cardboard cups and sugar starved 20 hours before the feeding experiment. The artificial blood meal for the survival assays was freshly prepared, consisting of rabbit erythrocytes, ATP (5 mM; Cayman Chemical, USA), FBS, and JNJ-A07 at 25 or 100 μM (exposed group) or the vehicle DMSO (vehicle control group, 1% final concentration of DMSO), and offered to the mosquitoes using the Hemotek membrane feeding system (Hemotek, UK). For the fecundity assays, the blood meal consisted of rabbit erythrocytes, ATP (5 mM; Cayman Chemical, USA), FBS, and JNJ-A07 at 2 or 25 μM (exposed group) or the vehicle (0.5% final concentration of DMSO). Mosquitoes were allowed to feed for 30 min, after which only fully engorged ones were selected and provided with 10% sugar solution. Notably, an additional control group was added for the fecundity assays which consisted of mosquitoes that received a mock blood meal containing rabbit erythrocytes, ATP (5 mM; Cayman Chemical, USA), FBS, and Dulbecco’s PBS.

For the survival assay, the mortality was recorded every day for each condition until 80% of the mosquitoes died. For fecundity measurement, each mosquito’s ovaries were dissected at day 4 after feeding, and the eggs developed per mosquito were counted. Notably, it has been reported previously that DMSO at concentrations up to 1% in the blood meal had no detrimental effect on mosquito life span, successive feedings, or physiological behavior (*45*).

### DENV oral infection and mosquito exposure to JNJ-A07

Two blood-feeding setups were used to evaluate the antiviral activity of JNJ-A07 in mosquitoes: (i) dose-response assays, in which the DENV-infectious blood meal was spiked with different concentrations of JNJ-A07, and (ii) consecutive blood-feedings, where the JNJ-A07–containing blood meal was given days before (pre-exposure prophylaxis) or following (post-exposure) the DENV-infectious blood meal.

#### Dose response

Female mosquitoes (5 to 10 days old) were starved 20 hours before oral infection, placed in cardboard cups, and transported to a biosafety level-3 facility. Mosquitoes were then offered an infectious blood meal spiked with JNJ-A07, consisting of fresh rabbit erythrocytes, ATP (5 mM), FBS, DENV-2 (5 × 10^6^ PFU/ml), and JNJ-A07 (final concentration ranging from 0.005 to 25 μM, exposed group) or the vehicle (maximum 0.5% final concentration of DMSO). Mosquitoes were fed for 30 min using the Hemotek system and then sorted while cold anesthetized. Only fully engorged females were selected, kept at 28°C and fed with 10% sugar solution for further sample collection at several time points pi.

#### Consecutive blood-feedings

Female mosquitoes were prepared as described above. In a pre-exposure prophylaxis setup, mosquitoes were offered a blood meal consisting of fresh rabbit erythrocytes, ATP (5 mM), FBS, and JNJ-A07 (2 μM, exposed group) or the vehicle (maximum 0.5% final concentration of DMSO). Following 30 min of feeding, fully engorged females were selected and provided with 10% sugar solution. An oviposition cup was supplied to the blood-fed females for egg laying. Female mosquitoes regain their host-seeking behavior starting at 12 hours after oviposition (*46*). Oviposition was observed in the mosquitoes at days 4 and 5 after blood meal; thus, the mosquitoes were offered a second blood meal 6 days after the first feeding. The eggs produced by the blood-fed mosquitoes were disinfected by immersion in Virkon S (Lanxess, AG, Germany) and carefully discarded. The second blood meal, consisting of fresh rabbit erythrocytes, ATP (5 mM), FBS, and DENV-2 (5 × 10^6^ PFU/ml), was given to the JNJ-A07– or vehicle-exposed mosquitoes. Following 30 min of feeding, engorged females were selected and kept at 28°C with access to a 10% sugar solution until sample collection at day 7 pi.

In the post-exposure setup, the order of the blood meals offered to the mosquitoes was inversed to the pre-exposure prophylaxis one. In brief, female mosquitoes were fed first with a DENV-infectious blood meal and allowed to lay their eggs in an oviposition cup. At 6 days pi, the mosquitoes were offered a second blood meal containing JNJ-A07 (2 μM) or the vehicle (maximum 0.5% final concentration of DMSO) and blood-fed mosquitoes were kept at 28°C with access to 10% sugar solution until sample collection at 13 days pi (7 days after exposure to JNJ-A07).

### Salivation and tissue dissection

At the selected time points pi, females were cold-anesthetized and forced salivation was performed to obtain saliva from each mosquito. In brief, wings and legs were removed from each mosquito using forceps. The proboscis of each mosquito was inserted in a tip filled with 20 μl of FBS for 1 hour. Next, each tip was collected, and the content was diluted with 40 μl of minimum essential medium supplemented with 2% FBS and 1% Pen-Strep. Following the forced salivation, each mosquito head was separated from the body. The head, wings, and legs of each mosquito were placed in homogenization tubes containing 2.8-mm ceramic beads (Precellys, Bertin Technologies, France) and 300 μl of PBS. The mosquito body was placed in a separate homogenization tube containing 300 μl of PBS.

All samples collected (saliva; body; head, wings, and legs) were stored at −80°C until further processing. Notably, the dissection forceps were disinfected in between samples by soaking in Virkon S (Lanxess, AG, Germany) followed by 70% ethanol to avoid cross-contamination.

### Sample analysis

Mosquito bodies, heads, wings, and legs were homogenized using bead disruption at 7800 rpm for 1 min (Precellys Evolution, Bertin Technologies, France). The homogenate was spun down at 8000 rpm for 1 min. The supernatant was transferred to a 0.8-μm Vivaclear Mini filter (Sartorius, Germany) and then centrifuged at 10,000 rpm for 2 min. The filtered homogenate was used in plaque assays (described above in this section) or focus-forming assays (FFAs) to detect infectious virus.

The FFA was used to determine the presence of infectious DENV-2 in saliva; body; head, wing, and leg samples. C6/36 cells were seeded at a density of 1 × 10^5^ cells per well in a black 96-well tissue culture plate (Greiner, Austria). One-day confluent monolayers of cells were inoculated with the corresponding samples, and the inoculum was incubated for 2 hours at 28°C, without CO_2_. After the inoculum was removed, 0.8% CMC overlay diluted in L-15 medium [supplemented with 1% Hepes, 1% nonessential amino acids (NEAA), 1% Pen-Strep, and 2% FBS] was added to each well. Cells were incubated for 7 days, after which monolayers were fixed by adding 4% paraformaldehyde on top of each well. After 1 hour of fixation, the overlay mixture was discarded, and the wells were carefully washed with PBS. The plates were stored at 4°C until the immunofluorescence assay was performed. Cells were incubated with 0.5% Triton (Sigma-Aldrich) in PBS at room temperature for 15 min, after which the cells were rinsed three times with wash solution [0.05% Tween 20 (Sigma-Aldrich) in PBS]. Next, the primary anti-DENV complex antibody clone D3-2H2-9-21 [1:500 diluted in block buffer (3% bovine serum albumin, 0.2% Tween 20, and 2% FBS); MAB8705; Sigma-Aldrich] was added and incubated at 37°C for 1 hour. The cells were rinsed three times with wash solution before incubation with the secondary antibody at 37°C for 1 hour: goat anti-mouse immunoglobulin G Alexa Fluor 594 (1:500 diluted in block buffer; A-11005; Invitrogen, Thermo Fisher Scientific). The cells were rinsed three times with wash solution, and then 4′,6-diamidino-2-phenylindole (DAPI) was added (final concentration of 100 nM) and incubated at room temperature for 15 min. Last, the cells were rinsed three times with wash solution, PBS was added, and the plates were stored at 4°C until imaging.

### Plate imaging and analysis of FFA

Images were acquired using an Operetta CLS system (Revvity, USA). The entirety of a well was imaged by capturing four fields per well with a 5× objective. The low magnification setting was selected to reduce data storage while maintaining qualitative images for downstream image analysis. Two separate channels were selected for image acquisition, the DAPI-stained nuclei were excited with a 355- to 385-nm light-emitting diode source, and emission was detected with a 430- to 500-nm filter, while the DENV-2 positive signal was excited at 530 to 560 nm, and emission was captured at 570 to 650 nm.

Image analysis was performed using the Harmony software (Revvity, USA). In-house algorithms were designed to count viable nuclei as an approximate for the cell count. In addition, infected cells were defined as Alexa Fluor 594–positive signals within viable nuclei.

### Concentration-time course of JNJ-A07

Female mosquitoes (5 to 10 days old) were fed with a JNJ-A07–spiked artificial blood meal consisting of rabbit erythrocytes, ATP (5 mM), FBS, and JNJ-A07 at 2 μM or the vehicle (maximum 0.5% final concentration of DMSO). The mosquitoes fed for 30 min, after which they were cold anesthetized, and only the fed females were transferred to cardboard cups for further collection. Mosquitoes from each condition were collected directly after feeding (day 0) and at days 1, 3, 5, and 7 after feeding. During each time point, mosquitoes were euthanized by placing them at −20°C for 10 min. Each mosquito was weighed in an analytical balance XS105 (Mettler Toledo, Switzerland) and stored individually in 1.5-ml tubes at −20°C. During the processing of the samples, each mosquito was transferred to a 2-ml Precellys tube containing ceramic beads and 200 μl of extraction solvent solution [mixture of methanol:water (7:3, v/v) and 0.1% formic acid]. Following homogenization, the solution was spun down for 5 min at 15°C and 5000*g*. The supernatant was used for further analysis. The mosquitoes fed on the vehicle-containing blood meal were used to prepare the quality control samples (blank matrix). Liquid chromatography with tandem mass spectrometry (LC-MS/MS) analysis was carried out on a 6500+ Triple Quad (Sciex), which was coupled to an Ultra Performance Liquid Chromatography (UPLC) system (Acquity UPLC; Waters, Milford, USA).

### LC method

Samples were quantified on a reversed-phase LC column (Acquity BEH C18 1.7 μm, 2.1 × 50 mm; Waters). Mobile phase consisted of 0.1% formic acid (solvent A) and acetonitrile (solvent B). Chromatographic elution was obtained by a gradient elution (50% solvent A; 50% solvent B starting conditions; to 10% solvent A; 90% solvent B in 1.0 min; isocratic hold at 95% B for 0.30 min and re-equilibrate to 50% A and 50% B in 0.4 min) at a flow rate of 0.600 ml/min.

### MS method

The MS/MS operated in the positive-ion mode (electrospray ionization) and was optimized for the quantification of the compound. Multiple reaction monitoring (MRM) transitions were as follows: 579.1 > 326 (quantifier) and 579.1 > 348 (qualifier). Samples were quantified against calibration curves prepared to cover the concentration range of the study samples. The curves were prepared in the same matrix as the study samples.

### Modeling

A logistic model was used for the analysis of virus dynamics in mosquitoes, as described previously (*47*). It accounted for variation in three parameters (*K*, *B*, and *M*) of a logistic model. This model was used to model the probabilities of systemic mosquito infection over time, assuming a binomial distribution of systemic infection status

$$f\left[t,(K,B,M)\right]=\frac{K}{1+e^{-B\left(t-M\right)}}$$

In this equation/distribution, *t* is a given time point; *K* is the saturation level and represents the maximum proportion of mosquitoes with a systemic infection, and *B* is the slope factor and represents the maximum value of the slope during the exponential phase of cumulative function, scaled by *K*. Δ*t* = log(81)/*B* is the time required to rise from 10 to 90% of the saturation level; *M* is the lag time and represents the time at which the absolute increase in cumulative proportion is maximal. Data from five time points were recorded: 3, 5, 7, 11, and 14 dpi. Three doses and the vehicle control (DMSO) were considered. Moreover, the estimated parameters were incorporated into a stochastic agent–based model designed to simulate the unfolding of real-world arboviral epidemics, as described previously (*47*).

### Data analysis

All figures and statistical analyses were generated with GraphPad Prism v9.5.1 (GraphPad Software, USA), except for the figures corresponding to the modeling exercise, which were generated using R. IR was calculated as the proportion of blood-fed mosquitoes with infectious virus present in the body. DIR was the proportion of mosquitoes with a positive infection in the body that also had infectious virus in the head, wings, and legs. TR was the proportion of mosquitoes with a disseminated infection that also had infectious virus present in the saliva. IRs, DIRs, and transmission rates were statistically compared using Fisher’s exact test.
