# Supplementary material for: The antiviral JNJ-A07 significantly reduces dengue virus transmission by Aedes aegypti mosquitoes when delivered via blood-feeding
Source: Sci Adv. 2024 Nov 27;10(48):eadr8338. doi: 10.1126/sciadv.adr8338 (PMC11601208; doi:10.1126/sciadv.adr8338)
Supplement: Supplementary file 1 — Figs. S1 to S4 Table S1 [file sciadv.adr8338_sm.pdf]

Science Advances
AAAS

Supplementary Materials for

The antiviral JNJ-A07 significantly reduces dengue virus transmission by *Aedes aegypti* mosquitoes when delivered via blood-feeding

Ana L. Rosales-Rosas *et al.*

Corresponding author: Leen Delang, leen.delang@kuleuven.be

Sci. Adv. **10**, eadr8338 (2024)
DOI: 10.1126/sciadv.adr8338

This PDF file includes:

Figs. S1 to S4
Table S1

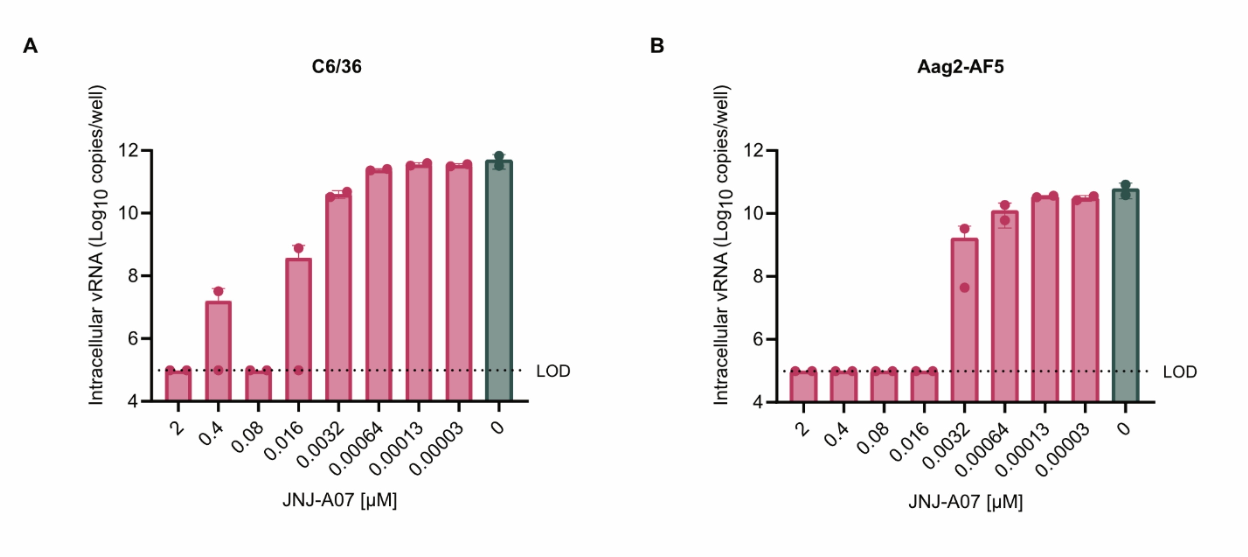

Fig. S1. *In vitro* activity of JNJ-A07 against DENV-2 in mosquito cell lines. Intracellular viral RNA (vRNA) levels were measured by qRT-PCR to evaluate the antiviral activity of JNJ-A07 against DENV-2 in *Aedes albopictus* (**A**, C6/36) and in *Aedes aegypti* (**B**, Aag2-AF5) derived cells. Data corresponds to two independent experiments, each with three replicates per concentration. The bar height represents the mean and the error bars correspond to the standard deviation. Each dot represents an independent experiment, each with three biological replicates per concentration tested. The dotted line displays the limit of detection (LOD) for the qRT-PCR assay.

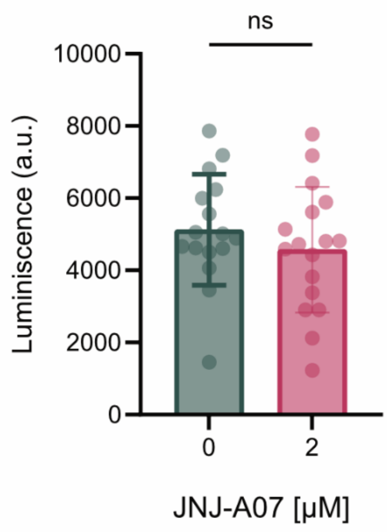

Fig. S2. Toxicity of JNJ-A07 in the *ex vivo* mosquito guts. The viability of the gut tissue was measured using ATP as indicator (CellTiter-Glo 3D). Luminescence readouts are displayed for guts incubated for 7 days with 0 (vehicle control, n=16) and 2 µM of JNJ-A07 (n=17). DMSO concentration was kept at 0.05% in both control and compound conditions. Each dot represents a biological replicate (one gut). The bar height represents the mean and the lines correspond to the standard deviation. Statistical significance was evaluated with an unpaired t-test (p=0.3431). Data shown corresponds to two independent toxicity experiments. a.u., arbitrary units.

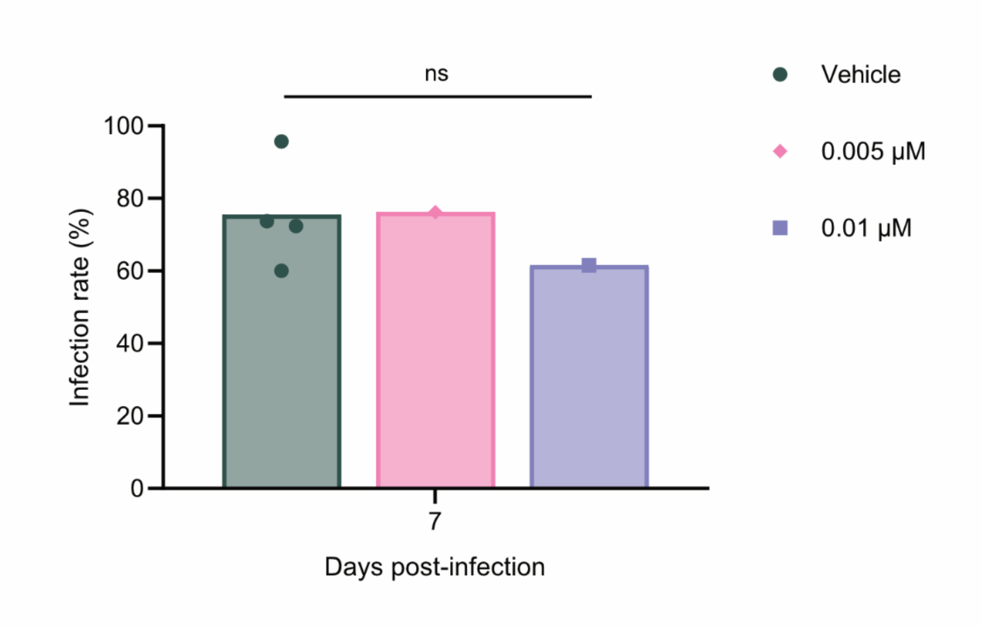

Fig. S3. No significant effect of low doses of JNJ-A07 on DENV infection rate in *Aedes* mosquitoes. DENV infection rates for mosquitoes that fed on a DENV-infectious bloodmeal spiked with JNJ-A07 at 0.005 (n=21), and 0.01 µM (n=26), and the vehicle (control group, n=91). Infectious virus was detected at day 7 post-infection by focus forming assay. Each symbol represents an independent blood-feeding experiment. The bar height represents the mean. Statistical significance was assessed with a Fisher's exact test ($p = 0.3371$).

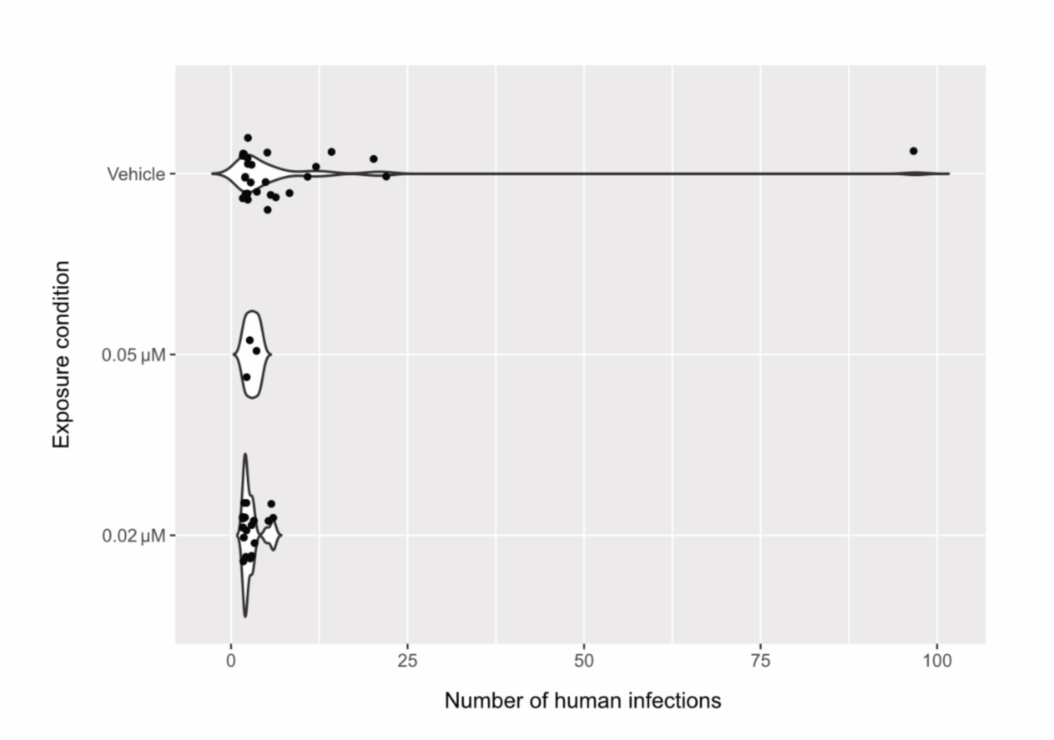

Fig. S4. Total number of humans that became infected during dengue outbreaks (< 100 humans infected) while using the stochastic agent-based model based on the empirical *K*, *B*, and *M* parameters for each condition tested.

Table S1. Prevalence of systemic infection. Dpi, days post-infection. *, data were extrapolated from available empirical data.

Condition	dpi	No	Positive	Prevalence	Low	Upper
Vehicle	3	44	1	0.0227273	0.0011875	0.1350905
Vehicle	5	30	3	0.1000000	0.0261883	0.2767632
Vehicle	7	91	24	0.2637363	0.1794080	0.3683350
Vehicle	11	43	8	0.1860465	0.0892174	0.3391789
Vehicle	14	21	3	0.1428571	0.0376430	0.3735664
0.02 µM	3	48	2	0.0416667	0.0072499	0.1542667
0.02 µM	5	42	3	0.0714286	0.0186305	0.2055446
0.02 µM	7	53	5	0.0943396	0.0352549	0.2142322
0.02 µM	11	27	1	0.0370370	0.0019364	0.2088918
0.02 µM	14	14	0	0.0000000	0.0000000	0.2676201
0.05 µM	3	35	0	0.0000000	0.0000000	0.1231534
0.05 µM	5	34	1	0.0294118	0.0015372	0.1705382
0.05 µM	7	34	0	0.0000000	0.0000000	0.1264034
0.05 µM	11	29	0	0.0000000	0.0000000	0.1456165
0.05 µM	14	21	1	0.0476190	0.0024908	0.2587351
2 µM	3	49	0	0.0000000	0.0000000	0.0905561
2 µM*	5	45	0	0.0000000	0.0000000	0.0979649
2 µM	7	50	0	0.0000000	0.0000000	0.0888758
2 µM*	11	47	0	0.0000000	0.0000000	0.0941149
2 µM*	15	47	0	0.0000000	0.0000000	0.0941149
